# Progesterone Has No Impact on the Beneficial Effects of Estradiol Treatment in High-Fat-Fed Ovariectomized Mice

**DOI:** 10.3390/cimb45050253

**Published:** 2023-05-03

**Authors:** Carlos H. Z. Talarico, Ester S. Alves, Jessica D. M. Dos Santos, Felipe G. S. Sucupira, Layanne C. C. Araujo, João Paulo Camporez

**Affiliations:** Department of Physiology, Ribeirao Preto School of Medicine, University of Sao Paulo, Ribeirão Preto 14049-900, Brazil

**Keywords:** menopause, ovariectomy glucose metabolism, metabolic syndrome

## Abstract

In recent decades, clinical and experimental studies have revealed that estradiol contributes enormously to glycemic homeostasis. However, the same consensus does not exist in women during menopause who undergo replacement with progesterone or conjugated estradiol and progesterone. Since most hormone replacement treatments in menopausal women are performed with estradiol (E2) and progesterone (P4) combined, this work aimed to investigate the effects of progesterone on energy metabolism and insulin resistance in an experimental model of menopause (ovariectomized female mice—OVX mice) fed a high-fat diet (HFD). OVX mice were treated with E2 or P4 (or both combined). OVX mice treated with E2 alone or combined with P4 displayed reduced body weight after six weeks of HFD feeding compared to OVX mice and OVX mice treated with P4 alone. These data were associated with improved glucose tolerance and insulin sensitivity in OVX mice treated with E2 (alone or combined with P4) compared to OVX and P4-treated mice. Additionally, E2 treatment (alone or combined with P4) reduced both hepatic and muscle triglyceride content compared with OVX control mice and OVX + P4 mice. There were no differences between groups regarding hepatic enzymes in plasma and inflammatory markers. Therefore, our results revealed that progesterone replacement alone does not seem to influence glucose homeostasis and ectopic lipid accumulation in OVX mice. These results will help expand knowledge about hormone replacement in postmenopausal women associated with metabolic syndrome and non-alcoholic fatty liver disease.

## 1. Introduction

Metabolic syndrome (MetSyn) can be characterized by a complex pathophysiological state which originates from several imbalances associated with caloric intake and energy expenditure. However, it is also affected by genetic/epigenetic factors and the predominance of a sedentary lifestyle over physical activity, among other factors such as food quality and composition, intestinal microbiota composition, and quality of life [1,2,3]. MetSyn can be described as a group of metabolic conditions that occur together and promote the development of physiological and pathophysiological disorders such as atherogenic dyslipidemia (elevated serum triglycerides (TAG), reduced high-density lipoprotein (HDL), and increased cholesterol), high blood pressure, cardiovascular diseases, and type 2 diabetes mellitus (T2DM) [1,2]. Generally, the critical component of this syndrome is the development of insulin resistance associated with obesity [1,2].

Global data on MetSyn are considered difficult to measure, and prevalence estimates variability based on the criteria used to define MetSyn [1]. However, since MetSyn is about three times more common than diabetes, it is estimated that the global prevalence may be about a quarter of the world’s adult population [1]. The prevalence of MetSyn has increased during the last three decades [1], but the understanding of the biology of the syndrome has also been expanded. Currently, several biological mechanisms are considered to cause insulin resistance in MetSyn [3]. Among the proposed mechanisms are endoplasmic reticulum stress [4], inflammation [5], and mitochondrial dysfunction [6], in addition to abnormal lipid metabolism [7] and ectopic accumulation [8].

MetSyn is mainly associated with the development of T2DM and is linked to characteristic dyslipidemia. Most obese individuals with T2DM and insulin resistance have an abnormal accumulation of TAG in their livers, characteristic of non-alcoholic fatty liver disease (NAFLD) [7,8]. NAFLD can be determined as the hepatic manifestation of MetSyn and is commonly associated with adjacent metabolic risk factors [7]. The progression of NAFLD can lead to hepatic steatosis, generally recognized as a benign disease, but can progress to non-alcoholic steatohepatitis (NASH). In addition to the presence of steatosis, NASH is typically characterized by lobular inflammation, hepatocyte ballooning, and perisinusoidal fibrosis [9,10]. Additionally, NASH can be a precursor to more severe liver diseases, such as cirrhosis and hepatocellular carcinoma [11]. Therefore, new approaches to the prevention and treatment of MetSyn are necessary to reduce the consequences of MetSyn, improving the quality of life of these individuals.

NAFLD is more prevalent in men than women of reproductive age [12]. However, in postmenopausal women, the prevalence of the disease becomes similar to that in men of the same age [12,13]. In recent decades, data from both clinical and experimental studies have revealed that endogenous steroid hormones such as estradiol (E2) contribute enormously to glycemic homeostasis [14,15]. Clinical studies also suggest the pivotal role of E2 in energy metabolism, since decreased estrogen levels during menopause are associated with increased visceral fat and, in turn, metabolic diseases such as insulin resistance, T2DM, and cardiovascular disease. In addition, women after menopause have an increased risk of glucose intolerance, insulin resistance, hyperlipidemia, and visceral fat accumulation [16,17,18]. Clinical studies have also shown that estrogen replacement therapy in postmenopausal women reduces the incidence of T2DM [19]. All of this evidence is closely related to NAFLD [12,20].

Progestogens are also a class of steroid hormones that bind to the nuclear progesterone (P4) receptor (PR) [21]. In addition to this, putative P4 membrane receptors PGRMC (P4 receptor membrane component) 1 and 2 have been identified in various human tissues, including the liver [22]. P4 is the body’s primary and most crucial progestogen and is essential in both female and male reproductive systems [23]. However, the literature regarding the effects of P4 on metabolic homeostasis is scarce, and few studies have focused on understanding such effects. It is also known that combined estrogen + progestogen therapy [24] is very effective in controlling the effects of estrogen deprivation during menopause. Because the excessive proliferation of endometrial cells leads to endometrial hyperplasia and cancer, which can result from estrogen-only therapy, progestogens have been administered continuously or sequentially in combination with estrogen to inhibit unwanted endometrial growth [25].

Based on what was observed in clinical studies with postmenopausal women and experimental studies with animals, and knowing that there is a significant gap in the literature on the knowledge of P4 effects on energy metabolism and insulin resistance and that most hormone replacement treatments in women are conducted with conjugate hormones E2 and P4, the main objective of this study was to investigate the potential effects of P4 on energy metabolism and insulin resistance in an animal model of menopause (ovariectomized mice) fed a high-fat diet (HFD), mimicking the effects of most risk factors associated with MetSyn and especially NAFLD.

## 2. Materials and Methods

### 2.1. Animals

Female mice with a C57BL/6J background were used. They were kept in a temperature-controlled room at 22 ± 2 °C with ad libitum access to food and water and submitted to a 12 h light–dark cycle (light from 6 a.m. to 6 p.m.). At eight weeks of age, the female mice were anaesthetized with isoflurane (~3%) and were ovariectomized (OVX). Then, they were randomly divided into 4 groups: OVX, OVX treated with E2 (OVX-E2), OVX treated with P4 (OVX-P4), and OVX treated with both E2 and P4 (OVX-E2-P4). To study the effect of chronic administration of E2 and/or P4, OVX mice were implanted subcutaneously with pellets releasing placebo or E2 or P4 (or both hormones) (0.05 mg/pellet of E2 for 60 days; 15.0 mg/pellet of P4 for 60 days; America’s Innovative Research, Sarasota, FL, USA) at the same time as the ovariectomy. A high-fat diet (HFD) (45% fat, D12451; Research Diets, New Brunswick, NJ, USA) was provided after ovariectomy and pellet implantation and continued for 6 weeks before the experiments. Since it was previously described that OVX mice and E2 treatment do not affect whole-body insulin sensitivity in regular chow-fed mice, it was decided to study just mice fed with HFD in this work [25].

All experiments carried out here were previously approved following the guidelines of the Ethics Committee of the Ribeirao Preto Medical School, University of São Paulo (CEUA—038/2021).

### 2.2. Glucose Tolerance Test

After 6 h of food restriction, mice were injected intraperitoneally (i.p.) with glucose (1 mg/kg body weight—10% dextrose). Blood samples for measuring glucose and plasma insulin were taken by tail bleeding at 0, 15, 30, 45, 60, 90, and 120 min after injection as previously described [26]. Plasma insulin was measured using a commercial ELISA kit (Mercodia, Winston Salem, NC, USA). The area under the curve (AUC) was calculated using the statistical software GraphPad Prism 9.0 in order to use it for statistical analysis.

### 2.3. Liver and Skeletal Muscle Lipid Measurement

After 6 h of food restriction, the animals were euthanized and the tissues were removed for lipid content analysis (liver and skeletal muscle—gastrocnemius). Tissue TAGs were extracted using the method of Bligh and Dyer [27] and measured using a TAG reagent (Bioclin, Brazil).

Aspartate Aminotransferase and Alanine Aminotransferase Measurement

Plasma was removed for analysis of liver enzymes (aspartate aminotransferase (AST) and alanine aminotransferase (ALT) using the commercial LabTest kit (LabTest, Belo Horizonte, Brazil).

### 2.4. RT-qPCR

The liver tissue was used for RT-PCR. The tissue was removed and 50 mg of the sample was homogenized in 1 mL of trizol (Life Technologies) for mRNA extraction. The sample was incubated for 5 min at room temperature (25 °C), and 200 µL of chloroform was added and incubated for 15 min at room temperature and centrifuged for 15 min at 2 °C at 12,000 rpm. The aqueous phase containing the RNA was separated; then, 500 µL of isopropanol was added and the sample was placed in a −20 °C freezer for 1 h. The sample was centrifuged for 10 min at 4 °C at 12,000 rpm and then underwent a washing process, the supernatant was discarded, and 1 mL of 75% alcohol was added and centrifuged for 10 min at 4 °C at 12,000 rpm; this step was performed 2 times in a row. The supernatant was discarded and the RNA went through a dissolution step, and 50 µL of RNAse-free water was added. The RNA concentration reading was evaluated at 260 nm, and the purity from the 260/280 nm ratio was analyzed with the nanodrop device (DeNovix). Then, the cDNA was prepared through a reverse transcription reaction (High-Capacity DNA kit, Applied Biosystems). A mix containing 10× RT buffer, 25× 100 Mm mixdNTP, 10× RT primers, reverse transcriptase, RNase inhibitor, and RNase-free water was prepared and added to the sample. The sample was taken to the thermal cycler. Gene expression was analyzed by RT-PCR (Rotor Gene Q—Qiagen) and SYBR Green fluorescent probe (Platinum^®^ SYBR^®^ Green qPCR Supermix UDG, Invitrogen).

The following primers for inflammatory markers were used: TNFα, IL-1β, TGFβ, and F4/80 (Table 1).

### 2.5. Statistical Analysis

Results were analyzed using GraphPad Prism version 9.0 (GraphPad Software, La Jolla, CA, USA). The minimum number of samples per group was defined by an n sufficient to perform the sample distribution analysis using the D’Agostino–Pearson omnibus normality test recommended by the GraphPad Prism 9.0 program. Results were expressed as means ± SD. Each experimental group had between 8 and 10 animals. Statistical analyses were performed using Bartlett’s test for homogeneity of variances followed by one-way analysis of variance (ANOVA) and Bonferroni multiple comparison test. The minimum acceptable significance level was *p* < 0.05.

## 3. Results

**Figure 1:**
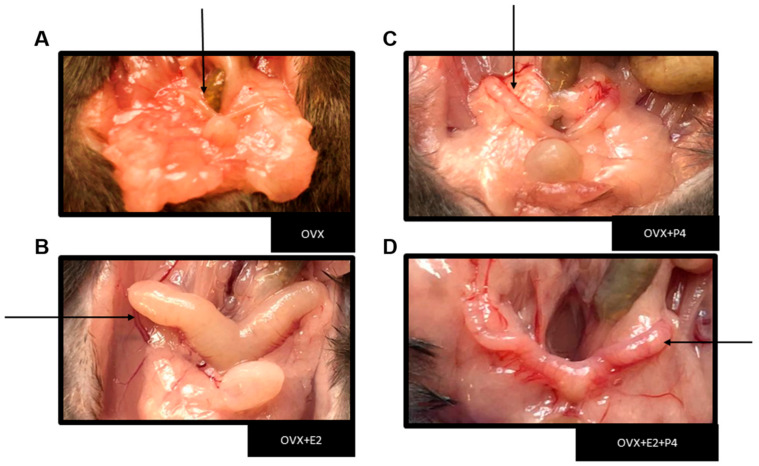
Representative image showing the uterus in each experimental group. Black arrows indicate the representative image of the uterus from each animal. (**A**) Image showing severe uterus atrophy in groups of ovariectomized mice and a large increase in peri-uterine adipose tissue. (**B**) Uterus with hormone replacement with E2. (**C**) Uterus with hormone replacement only with P4 (**D**) Uterus with hormone replacement with E2 + P4.

The performed ovariectomy was preceded by sedation and anesthesia through the monitored inhalation of isoflurane. After surgical recovery, the animals were fed a HFD for six weeks. The success of the ovariectomy was verified during the euthanasia of the animals, due to the absence of the ovaries and the intense atrophy of the uterine tubes (Figure 1) [24].

**Figure 2:**
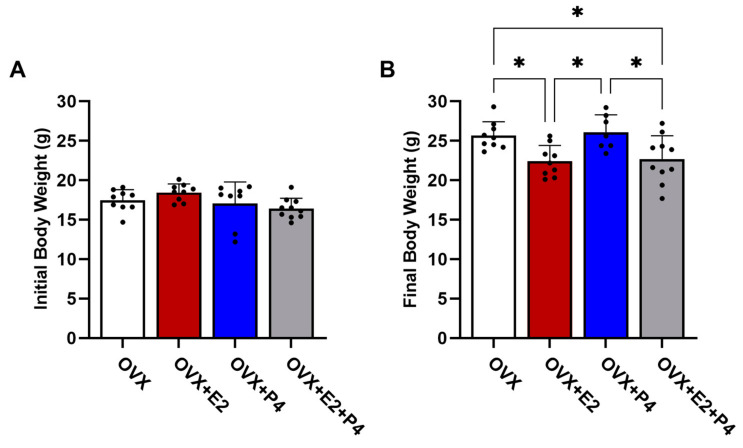
Initial (**A**) and final (**B**) body weight of animals before and after 6 weeks of HFD. Data are expressed as mean ± SD, * *p* < 0.05.

The animals were weighed before starting the diet and after six weeks on an HFD. Our results revealed that in the initial body weights, before the ovariectomy surgery, there were no statistically significant differences between the groups (*p* > 0.05) (Figure 2A). At the end of 6 weeks on an HFD, there were statistically significant differences in body weight in the OVX + E2 and OVX + E2 + P4 groups (*p* < 0.05) when compared with the OVX control group and the OVX + P4 group (Figure 2B).

**Figure 3:**
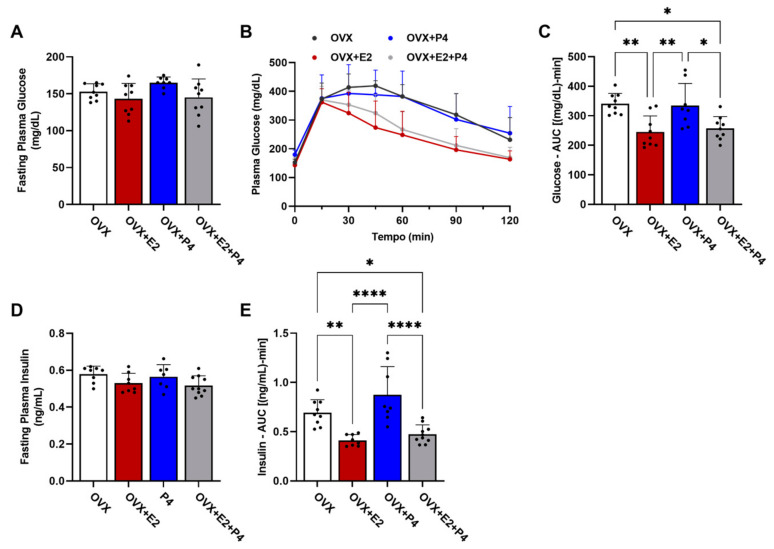
Glucose tolerance test and assessment of peripheral insulin resistance. (**A**) Fasting plasma glucose assessment. (**B**) Glucose tolerance test; plasma glucose was measured at 0, 15, 30, 45, 60, 90, and 120 min after I.P. of glucose. (**C**) AUC—glucose tolerance test. (**D**) Fasting plasma insulin. (**E**) AUC of plasma insulin during GTT. Data are expressed as mean ± SD, * *p* < 0.05, ** *p* < 0.01, **** *p* < 0.0001.

To characterize the metabolic phenotype, we performed the glucose tolerance test (GTT). This test measured changes in glucose levels in fasted animals (six hours) within a two-hour interval after the administration of 1 mg/kg of glucose, and the area under the curve (AUC) calculation was considered for statistical analysis. Measurements of insulin levels were also performed during this test, as well as the AUC calculation. Our results show that there were no statistical differences in baseline glucose levels between the groups (Figure 3A). The GTT test revealed differences between the groups (Figure 3B,C; OVX vs. OVX + E2; OVX vs. OVX + E2 + P4 and OVX + P4 vs. OVX + E2; OVX + P4 vs. OVX + E2 + P4), showing that the groups treated with E2 displayed improved glucose tolerance. Basal insulin did not show statistical differences between the groups (Figure 3D). Finally, plasma insulin levels during the GTT were also significantly lower in the OVX + E2 and OVX + E2 + P4 groups, revealing an indication of greater insulin sensitivity when compared with the OVX control and OVX + P4 groups. This was reflected in the AUC data for insulin (Figure 3E). There were no statistically significant differences between the OVX + E2 and OVX + E2 + P4 groups during the GTT (Figure 3E).

**Figure 4:**
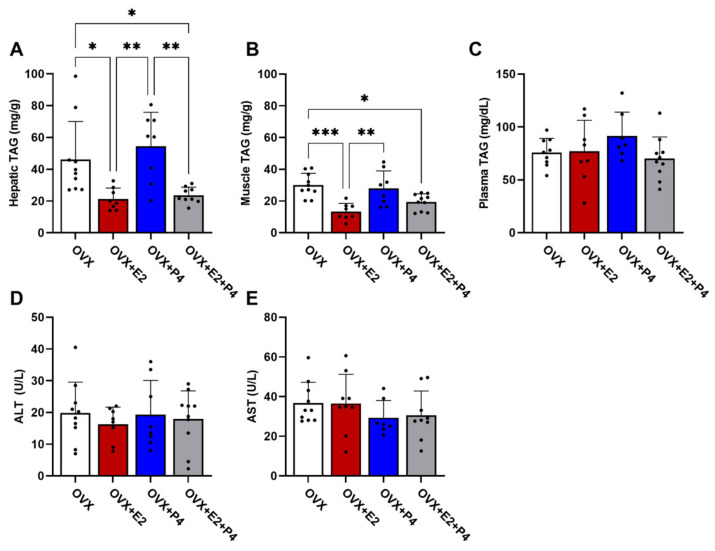
Analysis of TAG content. Analysis of lipid content extracted from the liver (**A**), muscle (**B**), and plasma (**C**). Analysis of the enzymes alanine aminotransferase (ALT) (**D**) and aspartate aminotransferase (AST) (**E**) in plasma. Data are expressed as mean ± SD, * *p* < 0.05, ** *p* < 0.01, *** *p* < 0.001.

There are several hypotheses of the mechanisms leading to insulin resistance. Among these hypotheses, the accumulation of lipids is one of the most important [8]. Thus, we understand the significance of evaluating the TAG content in the liver, skeletal muscle, and plasma. Our results demonstrated that the OVX + E2 and OVX + E2 + P4 animals presented a significantly lower TAG content in the liver compared with control OVX and OVX + P4 animals (Figure 4A). In skeletal muscle, OVX + E2 and OVX + E2 + P4 mice also displayed reduced TAG content (Figure 4B). There were no statistically significant differences in the plasma TAG concentration among the groups (Figure 4C).

AST and ALT are enzymes found primarily in the liver. These enzymes are used along with others to monitor the course of various liver disorders [28]. Our results did not indicate any significant difference between the groups in the analysis of these enzymes (Figure 4D,E).

**Figure 5:**
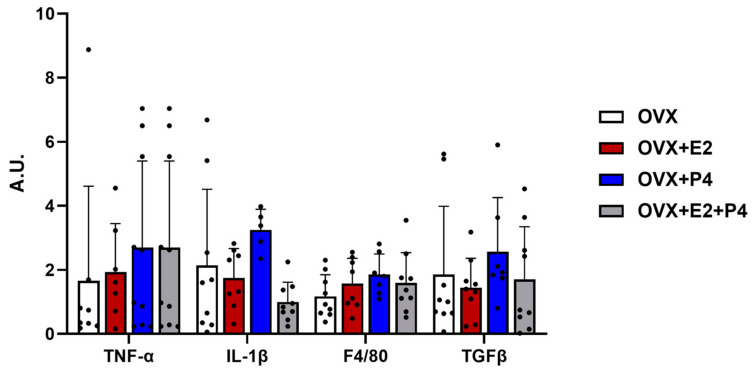
Gene expression of inflammatory and fibrotic markers. Relative levels of mRNA expression of genes related to inflammation and fibrosis did not show statistical differences. Data are expressed as mean ± SD.

Another hypothesis about the mechanisms related to insulin resistance is inflammation. In our study, we evaluated the expression by RT-PCR of anti-inflammatory cytokine markers such as transforming growth factor β (TGF-β) and pro-inflammatory cytokines such as interleukin 1 β (IL-1β), tumor necrosis factor-alpha (TNF-α), and F4/80 to check the evidence of infiltration and increased recruitment by macrophages [29,30]. Our results did not show significant differences between the described markers (Figure 5).

## 4. Discussion

Several studies have shown the effects of steroid hormones on metabolic homeostasis, most notably the effects of E2 [14,15,19,31,32,33]. However, the effects of the P4 hormone remain relatively controversial, since the literature on the topic is quite sparse [34,35]. In this work, we reported that E2 treatment exerted effects on body weight control in female OVX mice fed an HFD, confirming the effects of this hormone and corroborating the existing literature [13,15,31,36,37], as well as the role of estrogens and their recognized effect on glucose metabolism and insulin sensitivity [14,15,19,31,32,33].

Progesterone, administered to mice fed an HFD for six weeks, exerts no influence on body weight gain, showing less weight gain when combined with E2. Our results showed an improvement in glucose tolerance, insulin resistance, and TAG accumulation in the liver and muscle with the conjugated replacement of E2 + P4. This was not observed in animals with only P4 replacement, which was similar to the OVX control group, showing that P4 had little or no effect on glucose metabolism and insulin sensitivity in these animals.

Menopausal women may have increased body fat mass and visceral fat [34,36]. According to Lovejoy et al. [34], the increase in fat mass can be explained by reduced energy expenditure in postmenopausal women. These observations in women also fit experimental models of female OVX mice. In other studies, such as Rogers et al. [35] and previous studies by our group [13], OVX animals reduced total body O2 consumption and CO2 production, and energy expenditure, leading to increased body weight associated with increased fat mass. This study also revealed that although OVX mice were obese compared with OVX + E2 mice, their food intake was slightly lower than OVX + E2 mice, suggesting greater energy efficiency in these mice [13]. Chronic E2 replacement therapy corrected the reduction in energy expenditure and body O2 consumption, and this study also indicated a negative global energy balance for the treatment group in relation to a positive global energy balance for the OVX animals. Camporez et al. [13] also showed an oxidative reduction in the metabolism of white adipose tissue in OVX animals, associated with reduced expression of UCP-1, Cidea, and PRDM16, which may explain the decreased energy expenditure in these animals, since it is known that these proteins can improve the whole-body energy metabolism when highly expressed in this tissue [13]. All these published data can explain what was observed in our study, with OVX animals treated with E2 displaying reduced body weight and ectopic lipid content.

One of the major concerns with NAFLD is that ectopic lipid accumulation has been clearly linked to the development of hepatic insulin resistance and T2DM. Our results demonstrated that the OVX + E2 and OVX + E2 + P4 animals presented a significantly lower TAG content in the liver compared with OVX and OVX + P4 groups. In skeletal muscle, treatment with E2 and E2 + P4 also reduced the TAG content. Increased ectopic TAG content has been constantly related to increased diacylglycerol (DAG) content in the same tissues [8,33,38,39], which has been consistently associated with some isoforms of novel protein kinase C (PKCs) activation in muscle and liver in a state of insulin resistance. The activation of PKCθ associated with increased DAG content in skeletal muscle has been observed both in insulin-resistant rodents [32,40] and in humans [41]. In the liver, increased DAG content was associated with the activation of PKCε isoform both in experimental models of hepatic insulin resistance [40,41] and in humans with hepatic steatosis and insulin resistance [41], leading to impaired proximal insulin signaling. In hepatocytes, PKCε phosphorylates the insulin receptor (IR) in threonine, reducing its ability to auto-phosphorylate into tyrosine and trigger downstream insulin signaling, leading to hepatic insulin resistance [40]. Previous studies have already demonstrated the ability of E2 to reduce the accumulation of lipids and the activation of PKCε, which are observed in the animal model of menopause [15] and in HFD-fed male mice treated with E2 [31]. The results obtained in this work indicate that the hormonal replacement with P4 did not interfere with the ability of E2 to reduce ectopic lipid accumulation in muscle and liver.

Despite the observed differences in ectopic TAG concentrations (muscle and liver), we did not observe differences in plasma TAG levels. This is in line with what we observed earlier, where OVX mice fed an HFD did not show differences in plasma TAG levels when compared to SHAM and E2-treated OVX animals [13]. This was also observed in other works using different rodent models of obesity and insulin resistance [32,33,42]. These data are also in agreement with previously published works that demonstrate that wild-type mice, unlike humans, are resistant to hypertriglyceridemia induced by an HFD, and these animals may even present a reduction in plasma TAG levels with a very prolonged HFD feeding [43,44,45]. For this reason, there is a widespread use of KO animals (ApoE^-/-^ and LDLR^-/-^) for the study of atherosclerosis [46].

Changes in the profiles of pro-inflammatory and anti-inflammatory cytokines, such as IL-1β TNF-α and TGf-β, have also been observed in NAFLD [47,48,49]. In our study, in addition to the markers mentioned here, we evaluated other markers for inflammation, such as F4/80, to check for signs of infiltration and increased recruitment by macrophages and/or possible liver injury, as well as the analysis of liver enzymes AST and ALT [30,50]. The results revealed no changes between groups. It is important to emphasize that even the results of clinical studies in patients with NAFLD and overweight or obese are asymptomatic and have normal liver function tests [50]. The commonly observed biochemical pattern of increased inflammation and these enzymes is more associated with NAFLD progress and NASH [50].

Obesity observed in OVX mice was also associated with whole-body insulin resistance and impaired glucose tolerance [13,26]. Previous studies by Riant et al. [25] and Camporez et al. [13] already showed glucose intolerance and insulin resistance in OVX mice fed an HFD, and E2 replacement reversed these effects. Studies regarding P4 and insulin resistance are scarce; however, studies by our group [13] showed that intact female mice were protected from HFD-induced insulin resistance by endogenous E2 only in skeletal muscle, exhibiting hepatic insulin resistance, as were OVX mice [13]. SHAM animals clearly exhibit higher P4 concentrations than OVX and OVX + E2 mice. Camporez et al. [13] proposed that higher plasma P4 levels in SHAM mice could nullify the endogenous beneficial effects of E2 on insulin action in the liver. The possible mechanism by which P4 could prevent the effects of E2 would be to increase the expression of the enzyme estrogen sulfotransferase, a primary enzyme responsible for estrogen inactivation induced by increased P4 [51,52]. However, our observations showed that E2 + P4 conjugated replacement showed an indication of improvement in glucose tolerance and insulin resistance, similar to replacement with E2 alone. Another work by Lee et al. [51] showed that P4 could suppress gluconeogenesis after plasma insulin induction under normal conditions in a mouse model. However, P4 can increase blood glucose via gluconeogenesis in parallel with increases in Pgrmc1 expression, a novel membrane receptor for P4, and by increases in the key enzyme mediator of gluconeogenesis, phosphoenolpyruvate carboxykinase (PEPCK), in mice under conditions of insulin deficiency and insulin resistance, which may exacerbate hyperglycemia in diabetes, where insulin action is limited [51]. Nonetheless, it was not possible to observe any effect of P4 in OVX mice in our study.

Hormone replacement therapy is the most effective way to alleviate menopausal symptoms, such as vasomotor symptoms and genito-urinary menopause syndrome, and prevent bone loss and fracture [53]. However, the treatment must be individualized, and the patient’s history must be considered, considering the benefit–risk ratio for the treatment. According to The American Menopause Society, combined treatment of E2 with P4 should always be indicated in the case of women with a uterus, alleviating the possible carcinogenic effects of E2 on this organ [53]. In addition, there is no indication for hormone replacement therapy with progesterone alone, such as the treatment performed in our study. Our work aimed solely at the mechanistic study of possible effects of progesterone on glucose metabolism and insulin resistance, under no circumstance indicating the treatments carried out in our work should be applied to humans. Therefore, our results revealed that in HFD-fed mice, P4 replacement alone does not seem to influence glucose homeostasis and ectopic lipid accumulation, showing similar results to OVX animals. Conjugated E2 + P4 hormone replacement showed improved glucose tolerance, insulin resistance, and reduced accumulation of ectopic lipids, similar to replacement with E2. P4 showed little or no effect on these tests when associated with E2. Consequently, we believe these results will help expand knowledge about hormone replacement in menopausal women and its effects on metabolic homeostasis implicated in pathophysiologies such as MetSyn and NAFLD.

## Figures and Tables

**Table 1:** Primer sequences.

TNF-α	Sense Anti-sense	TCT TCT CAT TCC TGC TTG TGG C CAC TTG GTG GTT TGC TAC GAC G
IL-1β	Sense Anti-sense	GGC AGC TAC CTG TGT CTT TCC C ATA TGG GTC CGA CAG CAC GAG
F4/80	Sense Anti-sense	CCTGGACGAATCCTGTGAAG GGTGGGACCACAGAGAGTTG
TGFβ	Sense Anti-sense	CTCCCGTGGCTTCTAGTGC GCCTTAGTTTGGACAGGATCTG

## Data Availability

The data are not in a public archive database.
